# Comparative gene network analysis reveals drought resistance mechanisms in *Aegilops tauschii* genotypes

**DOI:** 10.1016/j.bbrep.2026.102663

**Published:** 2026-06-08

**Authors:** Mehran Falaknaz, Arash Noshadi, Mahsa Eshaghi, Alireza Mirzaeii, Abozar Ghorbani, Danial Kahrizi, Mahsa Rostami

**Affiliations:** a Agronomy and Plant Breeding Department, Faculty of Agricultural Sciences, University of Guilan, Rasht, Iran; b Oilseed Plants Research Institute, Razi University, Kermanshah, Iran; c Agronomy and Plant Breeding Department, College of Agricultural and Natural Resources, University of Tehran, Karaj, Iran; d Biotechnology Department, Faculty of Agriculture, Tarbiat Modares University, Tehran, Iran; e Agronomy and Plant Breeding Department, Faculty of Agriculture and Natural Resources Sciences, University of Mohaghegh Ardabili, Ardabil, Iran; f Nuclear Agriculture Research School, Nuclear Science and Technology Research Institute (NSTRI), Karaj, Iran

**Keywords:** *Aegilops tauschii*, Bioinformatics, Gene network, Drought tolerance, Protein-protein interaction network, Hub genes, Transcriptomics, *Cis*-regulatory elements

## Abstract

Wheat (*Triticum aestivum*) production faces growing threats from drought caused by climate change, which endangers global food security. *Aegilops tauschii*, the diploid ancestor of bread wheat, holds vital genetic resources for drought resistance. To decode the molecular basis of this resilience, we employed an integrated systems biology approach to analyze RNA-Seq data from drought-tolerant (XJ098) and drought-sensitive (XJ002) *Aegilops tauschii* genotypes. Differentially expressed genes (DEGs) under drought conditions were utilized to reconstruct genotype-specific protein-protein interaction (PPI) networks, facilitating the identification of central hub genes, functional modules, and *cis*-regulatory promoter motifs. Our network topology analysis revealed that XJ098 has a highly cohesive, modular network optimized for rapid stress signaling, in contrast to the decentralized, less cohesive network observed in XJ002. In XJ098, identified hub genes were significantly upregulated in coordinated pathways governing abscisic acid (ABA) signaling, cell wall remodeling (via expansins), osmotic adjustment (via aquaporins), and mitochondrial energy metabolism. Conversely, homologous hubs in XJ002 exhibited reduced connectivity and downregulation. Promoter analysis in XJ098 highlighted a significant enrichment of drought-responsive *cis*-regulatory elements, including ABRE, DREB, NAC, and MYB motifs, enabling a robust transcriptional response that was less pronounced in XJ002. Functional clustering indicated that XJ098 effectively coordinates these biological modules to enhance the biosynthesis of secondary metabolites, such as flavonoids, thereby fortifying antioxidant defenses and cellular integrity during water deficit. In summary, this research deciphers the complex regulatory networks governing drought tolerance in *A. tauschii*. The characterized hub genes and promoter motifs provide promising targets for functional validation and precision breeding, laying a crucial groundwork for developing climate-resilient wheat varieties.

## Highlights

• PPI analysis found hub genes (e.g., AET1Gv20557700) for drought tolerance in *A. tauschii.*
• XJ098 has drought-responsive motifs (ABRE, DREB) for transcriptional activation.
• XJ098 shows gene clusters for immune response, cell wall, and lipid metabolism.
• Candidate genes and networks identified for wheat breeding against drought stress.

## 1 Introduction

Wheat (*Triticum aestivum*) is a crucial staple for global food security, providing about 20% of the world's calorie intake and supporting the livelihoods of over two billion people [1]. The sustainability of wheat production faces growing threats from abiotic stresses, particularly drought, which is becoming more frequent and severe due to human-caused climate change [2,3]. According to the Food and Agriculture Organization (FAO), drought-related yield losses in key cereal crops, including wheat, range from 15% to 30%, worsening food insecurity and economic instability in vulnerable areas [4].

The hexaploid structure of the wheat genome, including the D subgenome derived from its wild diploid ancestor *Aegilops tauschii*, introduces genetic redundancy and complexity. However, it also offers a valuable pool of genetic diversity that can be utilized for abiotic stress tolerance [5,6]. Recognized for its natural drought resilience, *Aegilops tauschii* carries unique alleles not found in modern cultivars, making it a crucial resource for wheat breeding [7,8].

Despite progress in wheat genomics and breeding, understanding drought tolerance in hexaploid wheat remains challenging due to complex genomics and polyploidy, which make it difficult to identify gene-trait associations [9]. Therefore, studying *Aegilops tauschii* offers a simpler yet relevant system for identifying genetic factors influencing drought response, thereby supporting breeding efforts. Abiotic stress responses involve highly interconnected gene regulatory networks with transcription factors, signaling pathways, and downstream effectors that coordinate physiological and molecular changes [10,11]. While traditional differential gene expression studies provide useful insights, they often miss the complexity and hierarchy of these networks. Integrative systems biology methods, especially protein-protein interaction (PPI) network analysis combined with gene co-expression and promoter motif analysis, are effective in pinpointing key “hub” genes that serve as master regulators of drought adaptation [[12], [13], [14]].

These hub genes, due to their key connectivity and regulatory roles, are promising candidates for functional validation and genetic enhancement. Additionally, the identification of promoter motifs uncovers *cis*-regulatory elements that influence the spatiotemporal expression of drought-responsive genes, providing insights into transcriptional control mechanisms vital for adaptive stress responses [15,16]. Recent pangenome analyses and comparative genomics studies have revealed extensive allelic variation and novel regulatory variants in *Aegilops tauschii* that are missing from cultivated wheat, emphasizing the importance of characterizing its drought-responsive gene networks [6,17]. However, studies that comprehensively integrate PPI network reconstruction, hub gene identification, functional enrichment, and promoter motif analysis across different *Aegilops tauschii* genotypes under drought conditions are still limited.

To address this gap, our study employs a multi-layered bioinformatics pipeline on two *A. tauschii* genotypes (XJ002 and XJ098) with different drought tolerances. We analyze genotype-specific PPI networks, identify key hub genes, and examine their regulatory motifs to uncover the molecular mechanisms behind drought adaptation. This work aims to deepen our understanding of plant responses to abiotic stress and identify genetic targets to speed up the development of drought-tolerant wheat varieties. With water scarcity and climate change posing increasing global challenges, creating drought-resistant crops is vital for sustainable agriculture and food security [2,4]. Using the natural genetic variation in *Aegilops tauschii* and advanced network-based bioinformatics methods offers a promising approach to ensure wheat production in the future.

## 2 Materials and methods

### 2.1 Data acquisition and gene selection

RNA-Seq data for two *A. tauschii* genotypes with contrasting drought tolerance (XJ098: tolerant; XJ002: sensitive) were obtained from a previous study by Zhao et al. (2020) [12]. RNA-Seq was performed on seedling leaves of the two accessions, with three biological replicates, under control and drought stress conditions. Raw FASTQ files (41.8–59.4 million reads, 101 bp) were processed with fastp to remove adapters and low-quality reads (Q20 > 96.97%). Clean reads were mapped to the Ae. tauschii reference genome using Hisat2 (version 2.0.5) with default settings, resulting in a mapping rate of 92.07–93.38%. Gene expression levels were quantified using StringTie with FPKM normalization, and differential expression analysis (DEGs) was performed with DESeq2 (q-value ≤0.05, |log2 FC| ≥ 1). The data are available in the NCBI BioProject under accession PRJNA482066. DEGs were identified using DESeq2 (v1.34.0) with cutoffs of |log2 fold change| ≥ 1 and a false discovery rate (FDR) adjusted p-value <0.05 [18,19]. DEG lists specific to each genotype were used for further analysis. The complete list of analyzed genes is included in Supplementary File S1.

### 2.2 Protein-protein interaction network construction

Protein-protein interaction (PPI) networks were created separately for each genotype using the STRING database (version 12.0, https://string-db.org) [14], which compiles known and predicted interactions from various sources, such as experimental results, computational predictions, and text mining. An interaction score of at least 0.150 (medium confidence) was set to include biologically relevant connections while keeping the network specific. Self-interactions and duplicate edges were removed. The resulting PPI networks were then imported into Cytoscape (version 3.9.1) [20,21] for visualization and additional analysis.

### 2.3 Hub gene identification

Hub genes were identified using the CytoHubba plugin (version 0.1) in Cytoscape. Four topological algorithms, Maximal Clique Centrality (MCC), Degree, Density of Maximum Neighborhood Component (DMNC), and Maximum Neighborhood Component (MNC), were used to rank nodes by centrality measures [22]. The top 20 genes from each algorithm were selected, and those that consistently ranked high across different methods were considered potential hub genes for each genotype. To improve robustness and minimize bias, the top-ranked genes from each algorithm were compared. Genes appearing in multiple rankings were identified as candidate hub genes, with the top 10 from each method further analyzed to confirm their significance across different algorithms. For hub genes designated as uncharacterized in the primary dataset, functional annotations and protein domains were manually curated and retrieved by searching their respective gene identifiers against the UniProt Knowledgebase (UniProtKB).

### 2.4 Network clustering

Network clustering was performed in Cytoscape using the CytoCluster plugin (version 2.1.0) with the Identifying Protein Complex Algorithm (IPCA) [22]. IPCA detects densely connected subgraphs by calculating edge weights based on the number of common neighbors and summing these weights for each node. A cluster cutoff of 10 was set to ensure that clusters contained at least 10 nodes.

### 2.5 Hub cluster identification and functional enrichment analysis

To identify 'hub clusters' enriched with hub genes, the clusters from IPCA were intersected with a list of hub genes identified by CytoHubba. Clusters containing a significant number of hub genes were classified as hub clusters, which are hypothesized to be key functional modules central to drought response. Functional enrichment analysis of these clusters was performed using STRING's integrated Gene Ontology (GO) tools and KEGG pathway databases. Significance was determined by hypergeometric tests with Benjamini-Hochberg correction; adjusted p-values below 0.05 indicated statistical significance.

KEGG pathway enrichment analysis was conducted to explore the functional features of gene clusters. Since KEGG classifications are not entirely species-specific and can include disease categories from human research, the enrichment results were carefully reviewed. In this study, biological interpretation was limited to plant-relevant molecular functions and conserved cellular processes linked to stress responses.

### 2.6 Promoter motif discovery and cis-regulatory element identification

The 1 kb upstream promoter regions of the identified hub genes were retrieved from Ensembl Plants release 58 (May 2025) using the BioMart data mining tool [23]. De novo motif discovery was performed with MEME Suite (version 5.4.1), setting motif widths between 6 and 15 base pairs and using significance thresholds of p-value and E-value <0.01 [15]. Discovered motifs were then compared to known transcription factor binding profiles in the JASPAR CORE 2022 database via Tomtom (version 5.4.1), with only matches having q-values <0.05 retained to ensure biological relevance [24].

### 2.7 Software and computational environment

All analyses were conducted on a Linux-based high-performance computing system running Ubuntu 20.04. The software used included Cytoscape v3.9.1, CytoHubba v0.1, CytoCluster v2.1.0, STRING database v12.0, MEME Suite v5.4.1, Tomtom v5.4.1, and R v4.2.1 with Bioconductor packages such as DESeq2 v1.34.0. Parameter settings are detailed to facilitate reproducibility.

### 2.8 Computational validation of hub genes

To further verify the reliability of the hub genes identified from our RNA-Seq data, we conducted computational validation using an independent, publicly available dataset from a related drought-stress study. We compared the expression profiles of key hub genes to assess their consistency across datasets. Additionally, we performed literature mining to cross-validate the assigned biological roles of these genes.

## 3 Results

### 3.1 PPI network overview

**Fig. 1:**
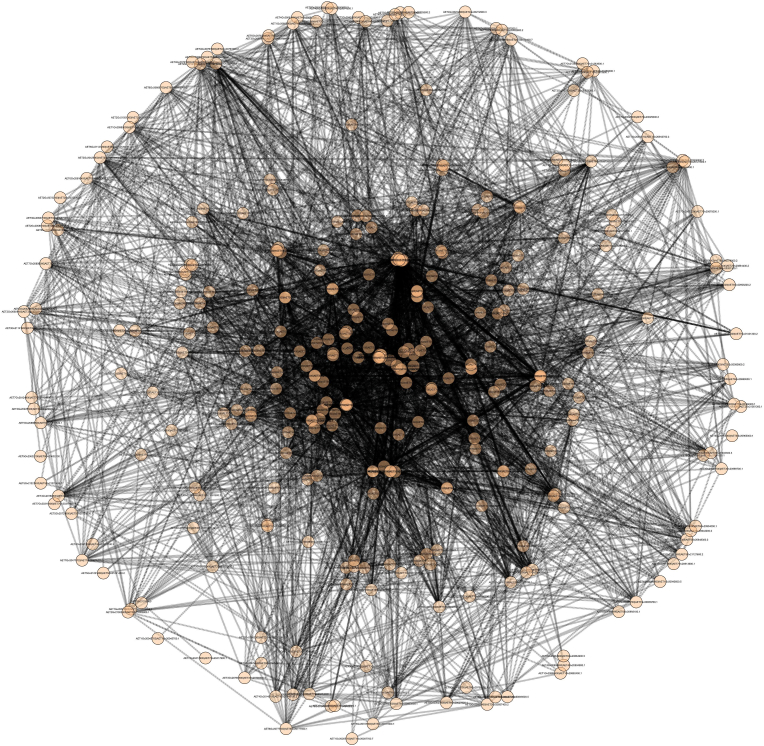
PPI Network of the up and down-regulated genes in XJ098 genotype involved in drought gene network using Cytoscape software.

**Fig. 2:**
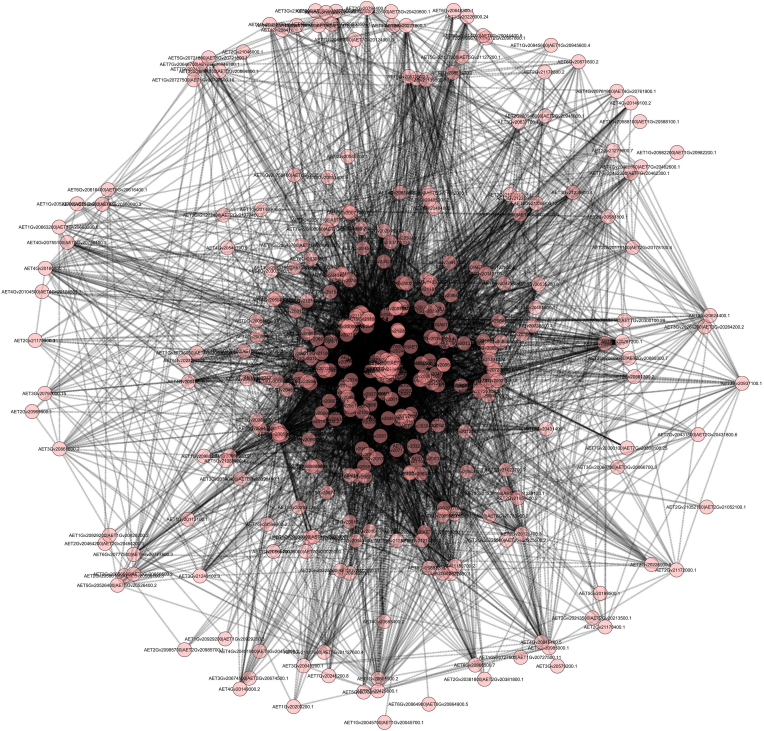
PPI Network of the up and down-regulated genes in XJ002 genotype involved in drought gene network using Cytoscape software.

Using STRING v12.0 and Cytoscape v3.9.1, PPI networks were reconstructed for drought-tolerant (XJ098) and drought-sensitive (XJ002) *Aegilops tauschii* genotypes based on drought-responsive DEGs. XJ098's network included 326 nodes and 4862 edges, with an average degree of 29.83, a clustering coefficient of 0.355, and moderate heterogeneity (0.735). These features suggest a highly modular, robust network optimized for rapid, coordinated stress signaling (Table 1, Fig. 1). In contrast, XJ002's network was larger and exhibited higher heterogeneity (348 nodes, 5488 edges, clustering coefficient 0.369, heterogeneity 0.777). Despite the higher number of edges, this increased heterogeneity suggests a decentralized network topology with less cohesive modularity, which could impair the rapid coordination of the drought response (Table 2, Fig. 2).

**Table 1:** Analysis of the network and the subnetwork gene in GC plus their known their know neighbors.

Number of nodes:	326
Number of edges:	4862
Avg. number of neighbors:	29.828
Network diameter:	5
Network radius:	3
Characteristic path length:	2.300
Clustering coefficient:	0.355
Network density:	0.092
Network heterogeneity:	0.735
Network centralization:	0.236
Connected components:	1

**Table 2:** Analysis of the network and the subnetwork gene in GC plus their known their know neighbors.

Number of nodes:	348
Number of edges:	5488
Avg. number of neighbors:	31.540
Network diameter:	5
Network radius:	3
Characteristic path length:	2.314
Clustering coefficient:	0.369
Network density:	0.091
Network heterogeneity:	0.777
Network centralization:	0.219
Connected components:	1

Networks of protein–protein interactions (PPIs) were created using the STRING database, applying a minimum interaction confidence score of 0.15 to include relevant but less characterized connections. These networks were further analyzed in Cytoscape to identify hub genes and vital modules based on network topology. While the low confidence threshold allowed for broader interaction detection, hub gene selection prioritized nodes with high topological centrality to ensure biological relevance.

### 3.2 Hub genes identification related to drought resistance

**Fig. 3:**
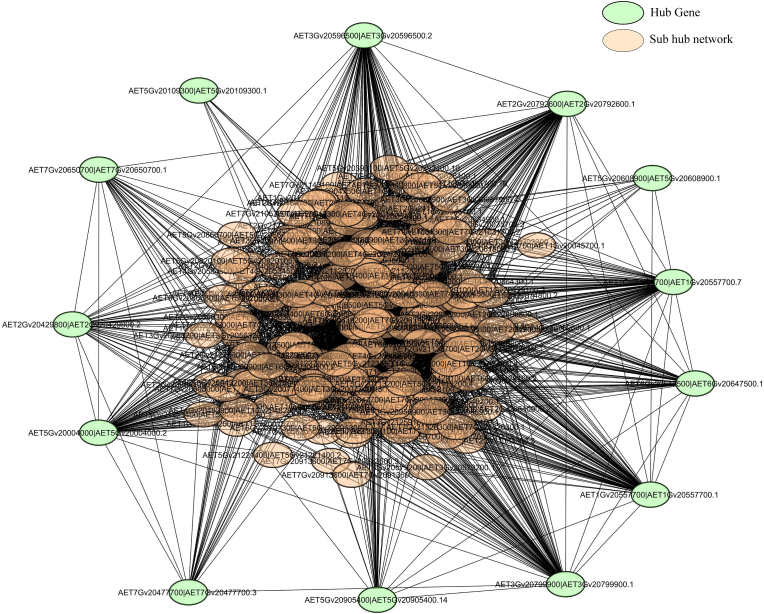
The sub network highlights the central hub genes in the XJ098 genome of *Aegilops tauschii*, depicted as nodes with numerous connections to surrounding green-colored genes, indicating their influence. The purple shading gradient represents gene expression levels, with darker shades indicating higher expression and potentially more significant roles in drought resistance. This visualization provides insights into the genetic interactions and molecular mechanisms at play.

**Fig. 4:**
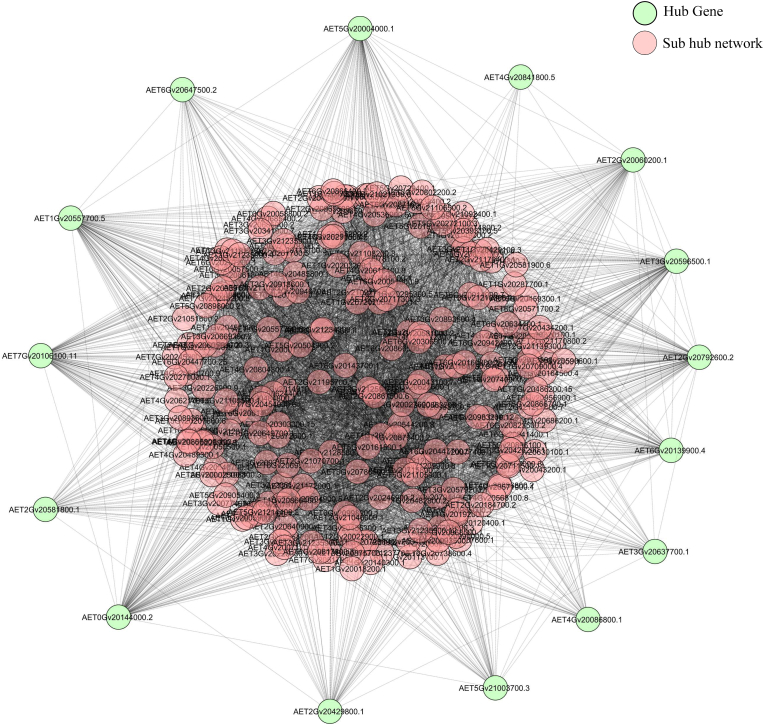
The network highlights the central hub genes in the XJ002 genome of *Aegilops tauschii*, depicted as green-colored nodes with numerous connections to surrounding genes, indicating their influence. The purple shading gradient represents gene expression levels, with darker shades indicating higher expression and potentially more significant roles in drought resistance. This visualization provides insights into the genetic interactions and molecular mechanisms at play. The hub genes are shown in the center in white with a yellow border.

Hub genes identified through four centrality metrics (MCC, Degree, DMNC, and MNC) act as key regulators in drought-response mechanisms (Table 1, Table 2; Fig. 3, Fig. 4). To ascertain the biological roles of genes initially identified as 'uncharacterized', their functional annotations were retrieved using the UniProt database. Notably, AET1Gv20557700 showed a 7.06-fold increase in expression (under drought stress compared to well-watered controls) and is functionally annotated as an ortholog of the AREB/ABF transcription factor family. ABA signaling is crucial for drought adaptation, facilitating stomatal closure via ion channel regulation and activating stress-responsive genes such as those encoding LEA proteins and osmoprotectants. Another critical hub gene, AET5Gv20004000, was upregulated 8.09-fold in XJ098 and encodes an Expansin-like CBD domain-containing protein. Expansins play a fundamental role in cell wall loosening and remodeling, which are vital processes for maintaining root elongation and cellular expansion under water-deficient conditions. Furthermore, AET6Gv20647500 exhibited a 7.99-fold upregulation and is annotated as an aquaporin, directly facilitating osmotic regulation and water transport to maintain turgor pressure. Additionally, AET3Gv20596500 (upregulated 5.63-fold) contains a FAS1 domain and is linked to mitochondrial function, supporting the energy metabolism required for stress responses.

### 3.3 Hub genes with reduced activity in XJ002

In contrast, several homologous hub genes were significantly downregulated or exhibited weak connectivity in the sensitive genotype, XJ002, under drought stress. For instance, AET2Gv20581800 (−5.32-fold) and AET3Gv20596500 (−4.15-fold) showed decreased expression, indicating compromised mitochondrial activity and energy metabolism. Similarly, AET2Gv20792600 (−4.0-fold) was downregulated, potentially disrupting the downstream transcriptional regulation of drought-responsive pathways. These deficiencies collectively reflect a weakened stress response network and contribute to the reduced drought tolerance observed in XJ002.

### 3.4 Pathways controlled by hub genes

Based on the functional annotation of the identified hub genes, drought adaptation in the tolerant genotype is driven by a highly coordinated and interconnected network of physiological pathways. The defense mechanism is primarily orchestrated by ABA-dependent signaling, specifically centered on the AREB/ABF transcription factor (AET1Gv20557700). This pathway plays a dual role by regulating stomatal closure to minimize transpiration and simultaneously activating the expression of protective genes that stabilize cellular structures. In conjunction with this initial signaling, the plant actively maintains its hydration status through precise osmotic adjustment and water transport. This process is directly facilitated by aquaporins, such as AET6Gv20647500, which ensure continuous water uptake and the preservation of cell turgor even under severe dehydration. To physically adapt to the water-deficient environment, the plant must also undergo critical cell wall remodeling. Key proteins, including the expansin-like AET5Gv20004000, mediate cell wall loosening to provide the structural flexibility required for sustained root elongation, allowing the plant to access deeper soil moisture. Ultimately, because orchestrating such a comprehensive defense network is highly energy-intensive, these processes are fundamentally supported by mitochondrial energy metabolism. Hub genes like AET3Gv20596500 are essential for maintaining mitochondrial integrity and ensuring consistent ATP synthesis, thereby fueling the active components of the plant's overall drought resilience strategy.

### 3.5 Functional clustering and network modules

**Fig. 5:**
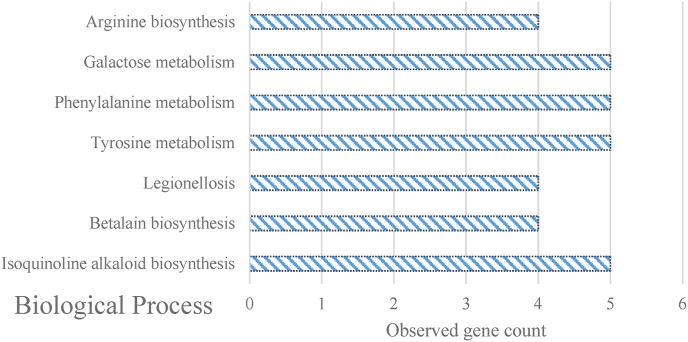
The role of the genotype of XJ098 in regulating processes dependent on chemical factors and related metabolic pathways.

**Fig. 6:**
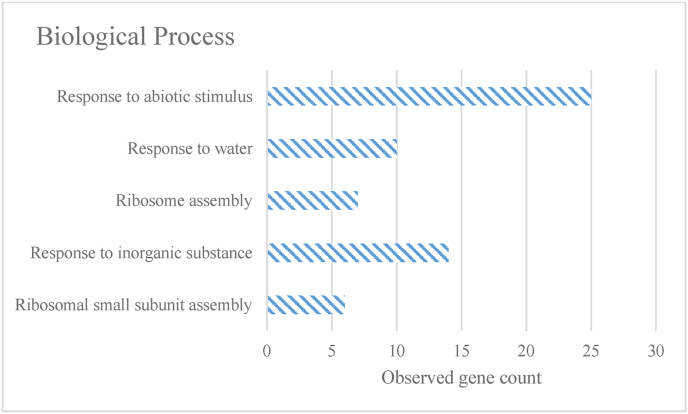
The role of the genotype of XJ002 in regulating processes dependent on chemical factors and related metabolic pathways.

Based on our IPCA clustering analysis (Table 9, Table 10; Fig. 5, Fig. 6), the hub genes were grouped into clearly defined, biologically cohesive modules. Looking specifically at the XJ098 genotype, the first cluster revealed an enrichment in plant defense-related signaling pathways (e.g., pattern-triggered immunity), highlighting the known crosstalk between biotic defense mechanisms and abiotic stress responses. The second cluster was characterized by glycoside hydrolase activity, which plays a vital role in restructuring the cell wall to improve flexibility and water retention. Additionally, the third and fourth clusters were linked to the biosynthesis of plant signaling lipids (such as jasmonates and phosphatidic acid), which are essential for repairing membranes and regulating stress signals. In sharp contrast, the modules associated with XJ002 were primarily driven by MAPK signaling and protein phosphorylation. While this points to an active stress-responsive signaling system, its actual downstream effects still require further experimental validation. Ultimately, these clustering patterns highlight the modular complexity of drought resistance. They demonstrate that XJ098 successfully coordinates its signaling and metabolic networks to mount a unified defense, whereas XJ002 exhibits a much more disconnected and fragmented response.

### 3.6 Promoter motif discovery and regulation

Promoter analysis identified conserved *cis*-regulatory elements in hub genes through the MEME Suite and the JASPAR CORE 2022 database (Table 7, Table 8). XJ098 promoters showed enrichment for ABRE, DREB, NAC, and MYB motifs, all linked to drought-responsive transcriptional regulation. These elements likely play a role in transcriptional responses during dehydration and ABA-related conditions. Conversely, XJ002 promoters mainly contained motifs related to general transcription and organelle function, without drought-specific elements, which may explain their weaker transcriptional activation. Overall, these results indicate that XJ098 has a more intricate transcriptional regulatory landscape, potentially contributing to its drought tolerance.

### 3.7 Metabolite and enzymatic responses

**Fig. 7:**
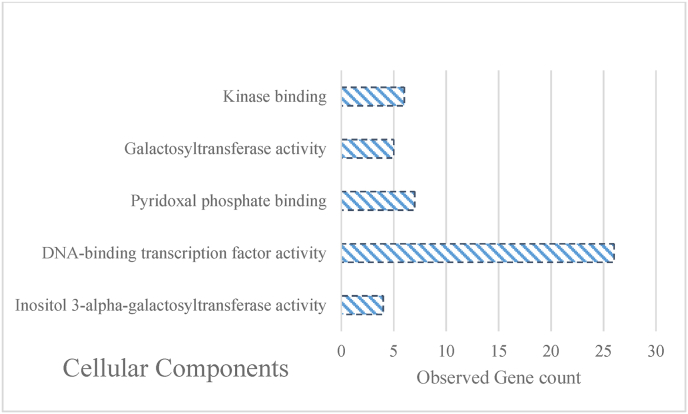
Enzymatic activities of the genotype of XJ098 and its involvement in catalytic processes and primary and secondary metabolic pathways.

**Fig. 8:**
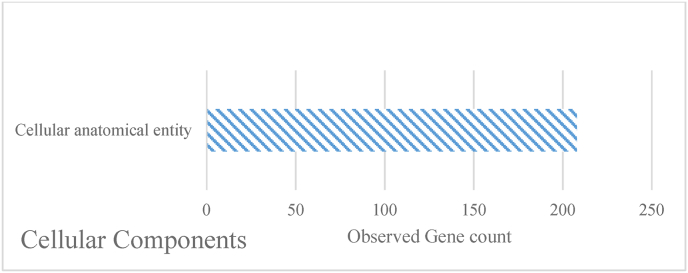
Enzymatic activities of genotype of XJ002 and its involvement in catalytic processes and primary and secondary metabolic pathways.

**Fig. 9:**
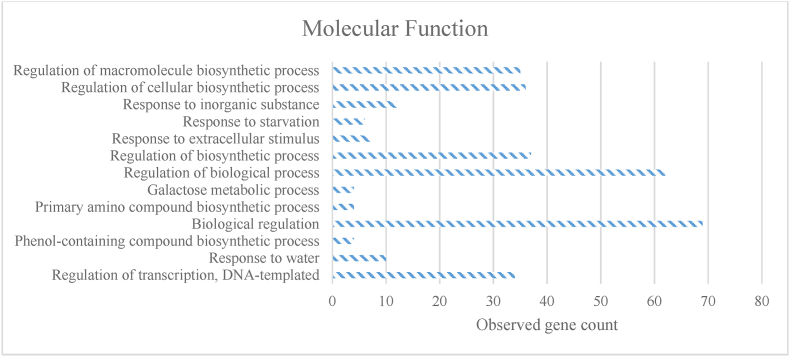
The impact of the genotype of XJ098 in response to secondary metabolites and its role in plant defense mechanisms against environmental stresses.

Enzymatic profiling revealed that XJ098 has higher activities in phenylpropanoid and flavonoid biosynthesis pathways, supporting antioxidant defenses and strengthening cell walls (Fig. 7). Elevated oxidoreductase activity helps maintain redox balance during stress. In contrast, XJ002 shows lower activation of these pathways, aligning with its reduced drought tolerance (Fig. 8). Additionally, the significant upregulation of genes encoding key enzymes in secondary metabolite biosynthesis suggests an enhanced accumulation of flavonoids and phenolics, thereby boosting drought resilience and defense mechanisms in XJ098 (Fig. 9).

## 4 Discussion

Drought tolerance is a complex trait governed by intricate gene regulatory networks that integrate hormonal, metabolic, and cellular signaling pathways. This study on *Aegilops tauschii* genotypes XJ098 (tolerant) and XJ002 (sensitive) highlights this complexity through PPI networks, hub gene prioritization, and promoter motif analysis, identifying key genetic and regulatory factors underlying drought resilience.

### 4.1 Network topology and functional modularity

Our analysis showed that the drought-tolerant XJ098 genotype has a PPI network with higher modularity and centralization compared to the sensitive XJ002 genotype (Table 1, Table 2, Table 3, Table 4, Fig. 1, Fig. 2). This aligns with recent studies in *Oryza sativa* and *Zea mays*, where increased network modularity was linked to enhanced abiotic stress tolerance, supporting more efficient signaling and coordinated gene regulation during stress [25,26].

**Table 3:** Analysis of the Hub-network and the subnetwork gene in GC plus their known their know neighbors.

Number of nodes:	207
Number of edges:	3640
Avg. number of neighbors:	35.169
Network diameter:	4
Network radius:	2
Characteristic path length:	1.940
Clustering coefficient:	0.402
Network density:	0.171
Network heterogeneity:	0.577
Network centralization:	0.347
Connected components:	1

**Table 4:** Analysis of the Hub-network and the subnetwork gene in GC plus their known their know neighbors.

Number of nodes:	251
Number of edges:	4606
Avg. number of neighbors:	36.701
Network diameter:	4
Network radius:	2
Characteristic path length:	2.016
Clustering coefficient:	0.436
Network density:	0.147
Network heterogeneity:	0.641
Network centralization:	0.283
Connected components:	1

### 4.2 Hub genes as master regulators of drought response

Among hub genes, AET1Gv20557700 emerged as a key ABA-responsive transcription factor with notable upregulation in XJ098 (7.06-fold) and strong activation in XJ002 (6.94-fold) (Table 5, Table 6). This TF is annotated as an ortholog of the ABF/AREB family, which is essential for ABA-mediated drought response by regulating stomatal closure and osmoprotective gene expression [27,28]. Similar functions for AREB TFs have been shown in wheat and barley, where overexpression improves drought tolerance by enhancing water-use efficiency and cellular protection [26]. The hub AET5Gv20004000**,** an Expansin-like protein, was markedly upregulated in XJ098 (8.09-fold) versus XJ002 (9.53-fold), highlighting the importance of ROS scavenging in drought defense. ROS detoxification hubs like this are well-documented in crops [29]. Our data support that tight regulation of ROS detoxification pathways is a conserved and critical mechanism in drought resilience.

**Table 5:** Hub genotype of XJ098 identified using DEGREE, MNC, MCC, and DMNC methods.

Hub Name in Ensemble	Rank	Algorithm	Fold change	Location
AET1Gv20557700	1,2,4,4	MNC,DMNC, DEGREE,MNC	7.0570	1D: 319885031-319885970
AET2Gv20429800	2	MMC	5.3373	2D: 170502457-170504288
AET2Gv20792600	1,2,2	MMC,DEGREE,MNC	4.5913	2D: 448881300-448882593
AET3Gv20596500	3,3	DEGREE,MNC	5.6335	3D: 347698928-347700462
AET3Gv20799900	5,5,5	DEGREE,MMC,MNC	4.7208	3D:469144703-469144943
AET5Gv20004000	3	MMC	8.0860	5D:2379348-2382958
AET5Gv20109300	3	DMNC	4.3010	5D:45542122-45543063
AET5Gv20608900	5	DMNC	4.1083	5D:45542122-45543063
AET5Gv20905400	1	DMNC	5.3164	5D:473910097-473990530
AET6Gv20647500	4	DMNC	7.9866	6D:347839059-347840812
AET7Gv20477700	2	DMNC	5.3427	7D:141457991-141459277
AET7Gv20650700	4	MMC	7.9330	7D:250837066-250837679

**Table 6:** Hub genotype of Xj002 identified using DEGREE, MNC, MCC, and DMNC methods.

Hub Name in Ensemble	Rank	Algorithm	Fold change	Location
AET0Gv20144000	5	Degree	3.64	jcf7190000162610:47602-51607
AET1Gv20557700	1,2,4	Degree, MNC	6.94	1D:31988503131988590
AET2Gv20060200	2	MCC	6.04	2D:13573331-13574433
AET2Gv20429800	3	MCC	4.05	2D:170502457170504288
AET2Gv20581800	4	MCC	−5.32	2D:31246635431246746
AET2Gv20792600	1,2	Degree, MNC	−4	2D:448881300448882593
AET3Gv20596500	3,3	Degree, MNC	−4.15	3D:347698928347700462
AET3Gv20637700	5	MCC	−4.33	3D:373540332373541152
AET4Gv20086800	1	DMNC	4.48	4D:21699033-21699778
AET4Gv20841800	2	DMNC	4.04	4D:514306700514308561
AET5Gv20004000	1	MCC	9.53	5D:2379348-2382958
AET5Gv21003700	4	DMNC	4.28	5D:504482847504488375

**Table 7:** Promoter analysis of the XJ098 gene using the MEME, TOMTOM, and GEMO databases.

Motif	Logo	Predictions	Top 5 specific predictions
AET1Gv20557700		20	MF structural constituent of ribosome BP translation MF nucleotide binding CC cytosolic large ribosomal subunit CC nucleolus
AET2Gv20429800		2	CC mitochondrion MF structural constituent of ribosome
AET5Gv20004000		12	CC mitochondrion BP translation MF structural constituent of ribosome MF nucleotide binding CC respiratory chain complex I
AET5Gv20905400		17	CC mitochondrion BP translation MF structural constituent of ribosome CC cytosolic large ribosomal subunit CC nucleolus
AET7Gv20477700		6	CC chloroplast MF transcription factor activity BP phenylpropanoid metabolic process BP DNA replication initiation BP regulation of transcription, DNA-dependent

**Table 8:** Promoter analysis of the XJ002 gene using the MEME, TOMTOM, and GEMO databases.

Motif	Logo	Predictions	Top 5 specific predictions
MA1222		4	MF transcription factor activity CC mitochondrion CC chloroplast stroma
MA1261		8	CC mitochondrion MF structural constituent of ribosome BP translation MF nucleotide binding CC ribosome
MA1522		8	CC chloroplast CC mitochondrion MF ATP binding CC nucleus MF transcription factor activity
MA1925		3	MF transcription factor activity CC endomembrane system BP regulation of transcription

**Table 9:** Overview of Clusters (Ranks 1 to 4) for Genotype Xj002 Derived from Cluster Analysis of Hub Gene sub network in the CytoCluster Application.

Rank	Nodes	Edges	Function
1	58	1044	MAPK signaling pathway - plant
2	52	956	MAPK signaling pathway - plant
			Protein phosphorylation
3	50	891	Protein phosphorylation
4	50	877	DNA-binding transcription factor activity

**Table 10:** Overview of Clusters (Ranks 1 to 4) for Genotype XJ098 Derived from Cluster Analysis of Hub Gene sub network in the CytoCluster Application.

Rank	Nodes	Edges	Function
1	49	823	Host–pathogen interaction–related signaling pathway
2	47	756	Glycoside hydrolase, family 16, active site, and EB1, C-terminal
3	47	754	Biosynthesis of E-series 18(S)-resolvins
4	46	757	Biosynthesis of specialized proresolving mediators

Note: Functional annotations were derived from KEGG-based enrichment analysis. Some pathway names may correspond to disease-associated categories due to conserved molecular components across species. Interpretation was restricted to plant-relevant biological functions.

Interestingly, the hub gene AET5Gv20004000, encoding an Expansin-like protein, was highly upregulated in both XJ098 (8.09-fold) and XJ002 (9.53-fold). Expansins are critical for cell wall loosening and root elongation. The strong expression in both genotypes suggests that structural remodeling of the cell wall to seek water is a basal response to drought. However, XJ098 successfully pairs this structural adaptation with superior osmotic regulation; for instance, the aquaporin-regulating hub AET6Gv20647500 was heavily upregulated in XJ098 (7.99-fold), acting as a key osmotic regulator that maintains cell turgor during dehydration [9]. The role of aquaporins in mediating water flow and drought tolerance has been confirmed in wheat, where aquaporin expression correlates with root hydraulic conductivity and drought adaptation [30]. This demonstrates that successful drought adaptation requires the synergistic action of both structural (expansins) and physiological (aquaporins) regulators.

Furthermore, proper energy balance is vital for activating these stress responses. AET3Gv20596500, involved in mitochondrial energy metabolism, was upregulated in XJ098 but significantly downregulated in XJ002. This highlights mitochondria's central role in sustaining ATP production under stress, aligning with studies showing mitochondrial dysfunction and energy depletion in drought-sensitive cultivars [31]. The transcription factor AET7Gv20477700, associated with MYB motifs and phenylpropanoid biosynthesis, was also highly expressed in XJ098. MYB TFs regulate pathways that produce flavonoids and lignin metabolites important for oxidative stress defense and strengthening cell walls [32].

### 4.3 Cis-regulatory motifs enable dynamic stress responses

Our promoter motif analysis identified an enrichment of ABRE, DREB, NAC, and MYB motifs in XJ098 hubs (Table 7, Table 8), which supports precise transcriptional regulation during drought conditions. The coexistence of ABA-dependent and ABA-independent motifs allows for complex regulation, enabling quick gene activation [13,33]. The lower presence of these motifs in XJ002 promoters might limit the transcriptional response, accounting for its weaker stress-induced gene activation. Similar motif enrichments have been observed in drought-tolerant wheat landraces, where promoter architecture dictates stress-induced gene expression [33,34].

### 4.4 Functional modules reflect integrated stress responses

Clustering analysis revealed that XJ098 hubs organize into cohesive modules involved in plant defense-related signaling, cell wall remodeling (glycoside hydrolase activity), and the biosynthesis of plant signaling lipids (Table 9, Table 10; Fig. 5, Fig. 6). This highlights the convergence of abiotic and biotic stress pathways [35], supporting a flexible and robust response to environmental challenges. Conversely, XJ002's hubs primarily cluster in MAPK signaling and phosphorylation modules, indicating initial stress detection without translating into effective downstream protective mechanisms. This pattern aligns with drought-sensitive rice genotypes, where early stress signaling alone fails to trigger structural and metabolic adaptation [35].

### 4.5 Metabolic reprogramming supports stress adaptation

XJ098 demonstrated increased oxidoreductase activity and secondary metabolite biosynthesis (Fig. 7, Fig. 8, Fig. 9), which are crucial for maintaining redox balance and structural integrity during drought stress [36]. The increased production of flavonoids and phenolics not only helps scavenge ROS but also reinforces cell walls, lessening damage during water scarcity.

The identified hubs and motifs serve as promising targets for functional validation and crop enhancement. Modifying the expression levels of key regulators, such as the AREB/ABF transcription factor (AET1Gv20557700) or water channels like aquaporins (AET6Gv20647500), through CRISPR or transgenic approaches could significantly boost drought tolerance in bread wheat. Additionally, utilizing synthetic promoters enriched with ABRE and DREB *cis*-elements might facilitate the precise stress-responsive expression of protective genes. Future studies should experimentally validate the in vivo functions of these hub genes, examine transcription factor binding dynamics, and incorporate epigenomic data to fully decode the regulatory networks governing drought resilience the dynamics of transcriptional regulation.

Network topology analysis revealed that the drought-tolerant genotype XJ098 possesses a PPI network with greater modularity and centralization than its sensitive counterpart XJ002. This finding is consistent with a study by Hao et al. (2022) ([37]), who demonstrated that drought-tolerant rice cultivars maintain highly interconnected gene modules that facilitate coordinated stress responses and recovery, a pattern directly mirroring our observations in XJ098.

## 5 Conclusion

This comprehensive study uncovers the intricate molecular mechanisms governing drought tolerance in *Aegilops tauschii* and offers a strategic avenue for improving wheat resilience to climate-induced water scarcity. The drought-tolerant genotype XJ098 features a highly modular and centralized PPI network, driven by critical hub genes that orchestrate ABA-dependent signaling, cell wall remodeling (via expansins), osmotic balance (via aquaporins), mitochondrial energy metabolism, and secondary metabolite biosynthesis. Collectively, these coordinated pathways significantly enhance resilience to water deficit. Furthermore, promoter analysis revealed significant enrichment of drought-responsive *cis*-elements, including ABRE, DREB, NAC, and MYB motifs, highlighting a multilayered transcriptional regulatory landscape that enables rapid and robust gene activation. While these systems biology insights provide a powerful computational framework, integrating empirical functional genomics such as CRISPR/Cas gene editing and promoter-reporter assays will be the next step toward establishing in vivo causality and fully translating these findings. The integration of multi-omics data, including proteomics and epigenomics, will also be beneficial for refining these network models. Ultimately, the identified hub genes and *cis*-regulatory motifs emerge as highly promising targets for precision breeding and synthetic biology. Harnessing the genetic diversity of *Aegilops tauschii* offers a strategic, actionable pathway to improve drought tolerance in bread wheat, safeguarding global crop production against escalating climate challenges.

## Authors’ contributions

M.F. conducted data identification and initial analysis. A N and A M conducted the initial writing of the article. The final version of the article was edited by M.F., A.GH., D.K., and M R. All authors approved the final manuscript.

## Ethics approval and consent to participate

Not applicable.

## Consent for publication

Not applicable.

## Funding

This research received no external funding.

## Declaration of competing interest

The authors of the manuscript entitled " **Comparative Gene Network Analysis Reveals Drought Resistance Mechanisms in *Aegilops tauschii* Genotypes** " namely **Mehran Falaknaz, Arash Noshadi, Mahsa Eshaghi, Alireza Mirzaeii, Abozar Ghorbani, Danial Kahrizi and Mahsa Rostami**, hereby solemnly declare that there exist no actual or potential conflicts of interest, financial, personal, or professional that could have inappropriately influenced the research, analysis, or interpretation of data presented in this manuscript.

## Data Availability

The original data were extracted from Zhao et al. Comparative transcriptome analysis of two *Aegilops tauschii* with contrasting drought tolerance by RNA-Seq. International Journal of Molecular Sciences, 21(10), 3595. doi: 10.3390/ijms21103595. PMID: 32438769; PMCID: PMC7279474.
